# Low lymphocyte-to-C-reactive protein ratio relates to high 1-year mortality in elderly patients undergoing hemiarthroplasty for displaced femoral neck facture

**DOI:** 10.1186/s13018-022-03406-9

**Published:** 2022-11-24

**Authors:** Jian Zhu, Xiaodong Cheng, Yonglong Li, Liping Bai, Zhongyi Su

**Affiliations:** 1 Department of Orthopaedic Surgery, Shanxi Bethune Hospital, Shanxi Academy of Medical Science, No. 99, Longcheng Street, Taiyuan, 030032 Shanxi Province China; 2 Department of Orthopaedic Surgery, The Third Hospital of Hebei Medical University, Shijiazhuang, 050051 China; 3 Department of Anesthesiology, Shanxi Bethune Hospital, Shanxi Academy of Medical Science, No. 99, Longcheng Street, Taiyuan, 030032 Shanxi Province China

**Keywords:** Elderly, Femoral neck fracture, Hemiarthroplasty, Mortality, Lymphocyte-to-C-reactive protein ratio

## Abstract

**Objective:**

Lymphocyte-to-C-reactive protein (CRP) ratio (LCR) is a novel biomarker for predicting poor prognosis in many diseases. This study aims to analyze the association between preoperative LCR and 1-year mortality in elderly patients with displaced FNF undergoing hemiarthroplasty.

**Methods:**

Between May 2017 and May 2019, a retrospective study including 364 elderly patients undergoing hemiarthroplasty for displaced FNF was performed. LCR was defined as the ratio of preoperative lymphocyte count to CRP level. The optimal cutoff value of LCR was determined by receiver operating characteristic curve, and all patients were categorized into low-LCR group and high-LCR group accordingly. The relationship between LCR and 1-year mortality was evaluated by using univariate and multivariate Cox regression analysis. Furthermore, the complications within 30 days after surgery, length of hospital stay, and perioperative red blood cell transfusion were also analyzed stratified by LCR.

**Results:**

A total of 47 patients (12.9%) died within 1-year follow-up after surgery. The optimal cutoff value for LCR was 30,560 (specificity 76.6% and sensitivity 63.4%). Low-LCR (≤ 30,560) group had a higher mortality rate than high-LCR group (23.53% vs. 5.21%, *P* < 0.001). In multivariate analysis, low LCR, hypoalbuminemia, and Age-Adjusted Charlson Comorbidity Index ≥ 6 were identified as independent predictors for 1-year mortality. Moreover, low level of LCR was associated with high rate of total complications (19.6% vs. 11.4%, *P* = 0.029), perioperative transfusions (37.9% vs. 27.0%, *P* = 0.027), and longer hospital stay (7.84 ± 2.40 vs. 7.30 ± 2.32, *P* = 0.031).

**Conclusions:**

The low level of preoperative LCR can effectively predict 1-year mortality and 30-day total complications after surgery in elderly patients with displaced FNF undergoing hemiarthroplasty.

## Introduction

Femoral neck fractures (FNF) in the elderly are associated with high rate of disability and mortality and more medical costs. In previous studies, the mortality has been reported to range between 12 and 37% within 1 year of the injury [1, 2]. With the aging of the general population and development of medical technology, the incidence of FNF will be increasing exponentially and the number has been estimated to be over 3.9 million worldwide by 2050 [3, 4]. Therefore, it is imperative to identify and correct the modifiable risk factors contributing to mortality to explore the optimal personalized therapy [1].

Recently, several immuno-inflammatory indices, such as neutrophil-to-lymphocyte ratio (NLR), lymphocyte-to-monocyte ratio (LMR), and C-reactive-protein-to-albumin ratio (CAR), have been reportedly correlated with non-oncological mortality in various surgical procedures, including hip fractures [5–10]. However, due to different characteristics, treatments, and mortality between femoral neck fractures and intertrochanteric fracture in elderly patients [11, 12], hip fractures should not be regarded as a single, homogeneous condition. Currently, little published literature exists detailing the association between theses immuno-inflammatory indices and mortality in elderly patients with FNF [13]. Moreover, as a novel biomarker of systemic inflammation, the lymphocyte-to-C-reactive protein ratio (LCR) has come to the fore and was reported to be correlated with reduced survival in patients with different diseases [14, 15]. However, as far as we known, there is no study available to date investigating whether the preoperative LCR could be used as a predictor of 1-year mortality in elderly patients with FNF.

Based on this, we performed a retrospective study to analyze the association between preoperative LCR and 1-year mortality in a cohort of elderly patients with displaced FNF undergoing hemiarthroplasty. Besides, the predictive value of LCR on complications after surgery was also analyzed.

## Materials and methods

### Study design, setting, and patients

This study was performed according to the Declaration of Helsinki and followed the recommendations of the STROBE guidelines. The Review Board of the Third Hospital of Hebei Medical University and Shanxi Bethune Hospital has reviewed and approved our research. All patients or their immediate family members provided written informed consents. From May 2017 to May 2019, consecutive elderly patients (age ≥ 65 years) undergoing hemiarthroplasty for displaced FNF in the orthopedics departments of the Third Hospital of Hebei Medical University and Shanxi Bethune Hospital were retrospectively studied. The inclusion criteria included: (1) aged over 65 years; (2) diagnosis of displaced FNF; (3) resulted from a fall from standing height or less; (4) time to surgery within 3 weeks; and (5) treated with hemiarthroplasty. On the other hand, the exclusion criteria were as follows: (1) pathological fractures; (2) with concomitant multiple fractures; (3) incomplete preoperative data; and (4) lost to follow-up.

### Clinical operations and post‑operative care

All hemiarthroplasties were performed by experienced surgeons and all patients received 2 g cefazolin prior to surgery. In case of hypersensitivity to cefazolin, clindamycin was used. A cemented or cementless prosthesis was implanted using posterolateral approach in a lateral decubitus position. Full weight-bearing mobilization as tolerated was allowed on the second day after surgery. Low molecular weight heparin was administered from admission to discharge for deep venous thrombosis (DVT) prophylaxis. Subsequently, oral rivaroxaban was prescribed for at least 1 month. Follow-up was undertaken every month in the first 3 months and then every 3 months until 1 year after surgery.

### Data extraction

The following data were collected and analyzed: patient age, gender, affected side, tobacco consumption, alcohol consumption, preoperative anemia, hypoalbuminemia, Age-Adjusted Charlson Comorbidity (AAC) Index, type of anesthesia, American Society of Anesthesiologists (ASA) score, prosthesis type, preoperative lymphocyte counts, C-reactive protein (CRP), preoperative and postoperative activity of patients, postoperative pain improvement, and mortality at 1 year postoperatively. Patients were categorized into two groups based on the median of age: ≤ 80 years and > 80 years. The anesthesia type was categorized as general or spinal/epidural anesthesia. Based on the total score of AAC Index for each patient, the study population was divided into three categories: 1–3, 4–5, and ≥ 6 points [16]. Venous blood samples were taken at admission. The definition of anemia was preoperative hemoglobin concentration < 110 g/L in women and < 120 g/L in men. Meanwhile, the definition of hypoalbuminemia was preoperative albumin < 35 g/L. The three-step analgesic ladder of the WHO [17] was used to document postoperative pain levels. Outcome parameters included the duration of strong opioid analgesic that was used after surgery and the level of analgesic was used at discharge. The postoperative activity of patients was evaluated as the capability of walking with a walker or crutches after surgery [18]. The LCR was defined as the ratio of preoperative lymphocyte count (number/µL) to CRP level (mg/L) [19, 20].

The data on mortality were obtained from medical records and telephone interviews. Besides, the data on red blood cell transfusion, length of stay, and 30-day complications postoperatively were also collected. Length of stay was defined as the duration from operation to discharge. The postoperative complications included urinary tract infection, wound infection, pneumonia, stroke, congestive heart failure, and deep vein thrombosis.

### Statistical analysis

Normal distribution was evaluated with the Shapiro–Wilk test. Continuous variables were expressed as mean ± standard deviation (SD) or median values (interquartile ranges [IQRs]). Differences between the low-LCR groups and high-LCR group were assessed with the independent Student’s *t* or Mann–Whitney U tests based on the data distribution. Categorical variables were showed as number and percentage (%) and were analyzed with Chi-square or Fisher’s exact test, as appropriate. The optimal cutoff value of LCR, CRP, and lymphocyte were determined by receiver operating characteristic (ROC) curve, when Youden index was maximum. The area under the curve (AUC) analysis was used to test significance of the ROC curve, with *P* < 0.05 as significance level. Univariate and multivariate analyses for 1-year mortality were analyzed with the Cox proportional hazard model. Survival of patient was estimated with the Kaplan–Meier curves and compared with the log-rank test. The statistical software used was SPSS Statistics 26.0 (IBM Corp., Armonk, NY, USA) and R software (version 3.6.5, R Foundation for Statistical Computing, Vienna, Austria). A two-sided *P* < 0.05 was regarded as statistical significance.

## Results

### Patient characteristics

**Fig. 1:**
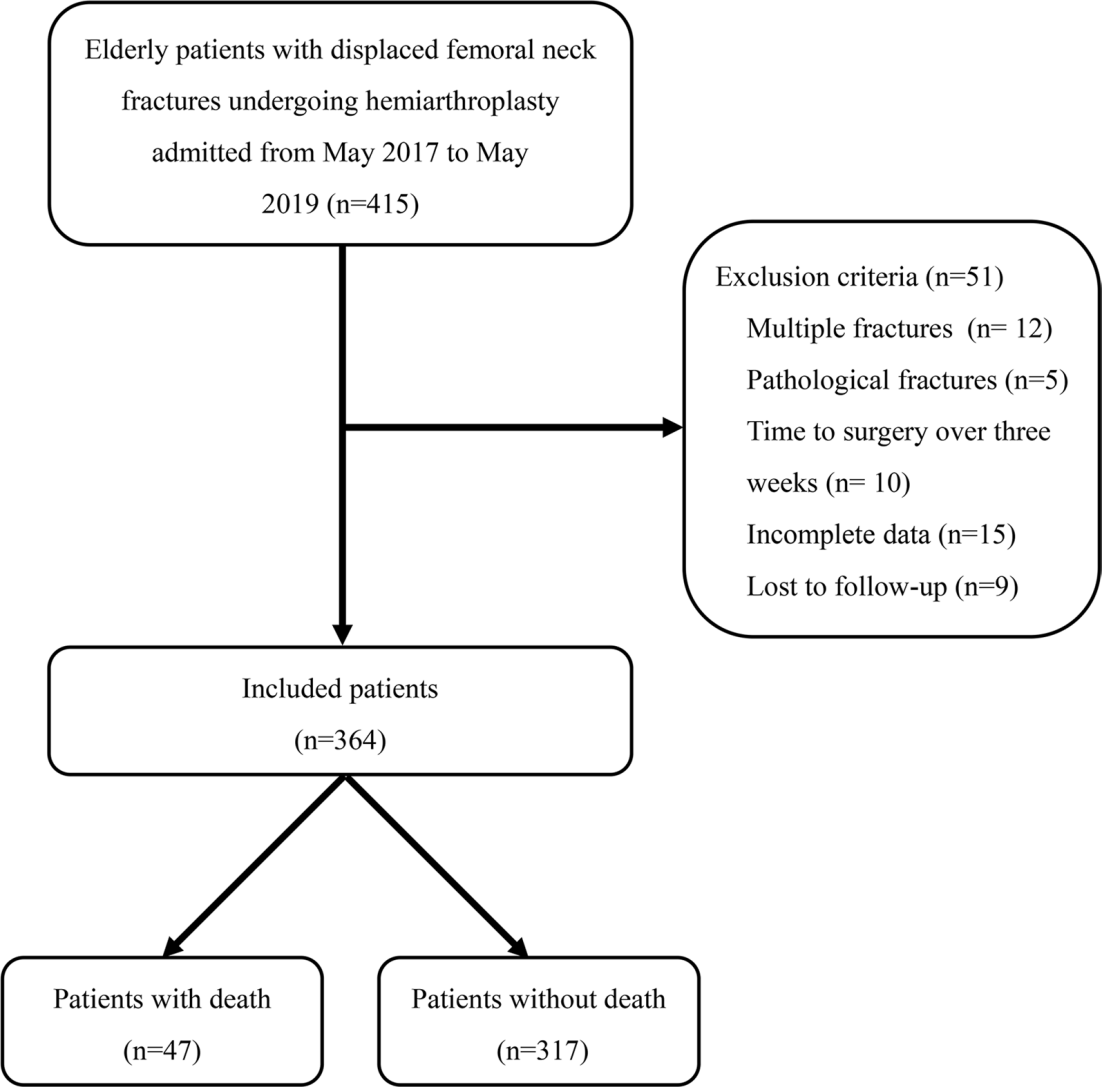
Flow diagram

As presented in Fig. 1, a total of 364 patients were included in this study, consisting of 114 males and 250 females, with a mean age of 79.5 (range, 65–99) years. Among of them, 47 cases (12.9%) died within 1-year follow-up after surgery. No perioperative mortality was observed. Preoperative anemia was observed in 31.6% (115) of the patients and hypoalbuminemia in 35.7% (130) of patients. With respect to AAC, 158 (43.4%) had low AAC scores (1–3), 132 (36.3%) had moderate AAC scores (4–5), and 74 (20.3%) had high AAC scores (≥ 6). In addition, 56.3% received general anesthesia, 47.8% classified as ASA class III–IV, and 58.2% underwent hemiarthroplasty with cement prosthesis.

### Identification of optimal cutoff values for lymphocyte, CRP, and LCR

**Fig. 2:**
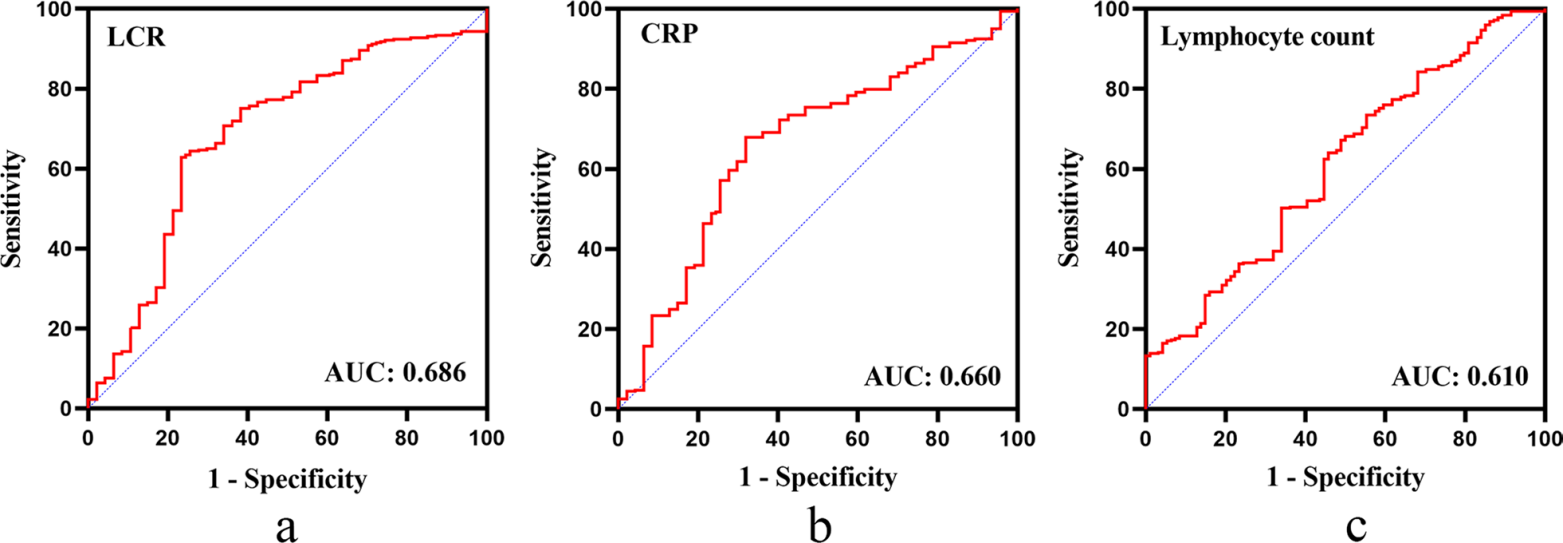
ROC curves analysis presenting predictive values of LCR, CRP, and lymphocyte count for 1-year mortality in elderly patients with displaced femoral neck facture undergoing hemiarthroplasty. *ROC* Receiver operating characteristic curves, *LCR* lymphocyte-to-C-reactive protein ratio, *CRP* C-reactive protein, *AUC* area under the ROC curve

To determine the most valuable biomarker for predicting 1-year mortality, the optimal cutoff value and corresponding ROC curve (AUC) were calculated. They were lymphocyte count: 0.925 (0.610); CRP: 39.92 (0.660); LCR: 30,560 (0.686). The details were showed in Table 1 and Fig. 2. As the AUC for LCR was larger than lymphocyte count and CRP, we further evaluated the predictive value of LCR for 1-year mortality.

**Table 1:** Comparison of the AUC between the LCR, CRP, and lymphocyte count

Variable	AUC	Sensitivity	Specificity	95% CI	*P* value
Lymphocyte count	0.610	0.735	0.447	0.523–0.697	0.015
CRP	0.660	0.681	0.678	0.576–0.743	< 0.001
LCR	0.686	0.634	0.766	0.600–0.772	< 0.001

*AUC* Area under receiver operating characteristic curve, *CI* confidence interval, *CRP* C-reactive protein, *LCR* lymphocyte-to-C-reactive protein ratio

### Relationship between baseline data and preoperative LCR

Table 2 showed the baseline data between the low-LCR group and high-LCR group. It can be seen that low LCR was closely correlated with previously established factors for poor prognosis in elderly patients with FNF, including older age (*P* = 0.037), anemia (*P* = 0.048), hypoalbuminemia (*P* < 0.001), and high score of AAC (*P* < 0.001). Similarly, the 1-year mortality was higher in the low-LCR group compared with the high-LCR group (*P* < 0.001).

**Table 2:** Baseline data according to LCR in elderly patients with displaced femoral neck facture undergoing hemiarthroplasty

Variable	Total (*n* = 364)	Low LCR (*n* = 153)	High LCR (*n* = 211)	*P* value
Age (median, years)				0.037
≤ 80, *n* (%)	204 (56.0)	76 (37.3)	128 (62.7)	
> 80, *n* (%)	160 (44.0)	77 (48.1)	83 (51.9)	
Gender				0.064
Female, *n* (%)	250 (68.7)	97 (38.8)	153 (61.2)	
Male, *n* (%)	114 (31.3)	56 (49.1)	58 (50.9)	
Affected side				0.185
Left, *n* (%)	185 (50.8)	84 (45.4)	101 (54.6)	
Right, *n* (%)	179 (49.2)	69 (38.5)	110 (61.5)	
Tobacco consumption				0.710
Yes, *n* (%)	15 (4.1)	7 (46.7)	8 (53.3)	
No, *n* (%)	349 (95.9)	146 (41.8)	203 (58.2)	
Alcohol consumption				0.882
Yes, *n* (%)	9 (2.5)	4 (44.4)	5 (55.6)	
No, *n* (%)	355 (97.5)	149 (42.0)	206 (58.0)	
Anemia				0.048
Yes, *n* (%)	115 (31.6)	57 (49.6)	58 (50.4)	
No, *n* (%)	249 (68.4)	96 (38.6)	153 (61.4)	
Hypoalbuminemia				< 0.001
Yes, *n* (%)	130 (35.7)	71 (54.6)	59 (45.4)	
No, *n* (%)	234 (64.3)	82 (35.0)	152 (65.0)	
AAC				< 0.001
AAC = 1–3, *n* (%)	158 (43.4)	38 (24.1)	120 (75.9)	
AAC = 4–5, *n* (%)	132 (36.3)	66 (50.0)	66 (50.0)	
AAC ≥ 6, *n* (%)	74 (20.3)	49 (66.2)	25 (33.8)	
Type of anesthesia				0.498
General, *n* (%)	205 (56.3)	83 (40.5)	122 (59.5)	
Spinal/epidural, *n* (%)	159 (43.7)	70 (44.0)	89 (56.0)	
ASA class, *n* (%)				0.809
I–II, *n* (%)	190 (52.2)	81 (42.6)	109 (57.4)	
III–IV, *n* (%)	174 (47.8)	72 (41.4)	102 (58.6)	
Prosthesis type				0.65
Cement, *n* (%)	212 (58.2)	87 (41.0)	125 (59.0)	
Cementless, *n* (%)	152 (41.8)	66 (43.4)	86 (56.6)	
Lymphocyte count (× 109/L)	1.16 (0.87–1.50)	0.98 (0.74–1.39)	1.18 (0.90–1.54)	0.015
CRP (mg/L)	28.23 (13.70–53.33)	49.72 (25.20–78.20)	25.46 (12.56–49.56)	< 0.001
LCR	44,304 (17,705–94,951)	18,597 (10,170–30,556)	48,764 (21,438–105,664)	< 0.001
1-year mortality				< 0.001
Yes, *n* (%)	47 (12.9)	36 (76.6)	11 (23.4)	
No, *n* (%)	317 (87.1)	117 (36.9)	200 (63.1)	

*LCR* Lymphocyte-to-C-reactive protein ratio, *AAC* Age-Adjusted Charlson Comorbidity, *ASA* American Society of Anesthesiologists, *CRP* C-reactive protein

### Relationship between clinical variables and 1-year mortality

**Fig. 3:**
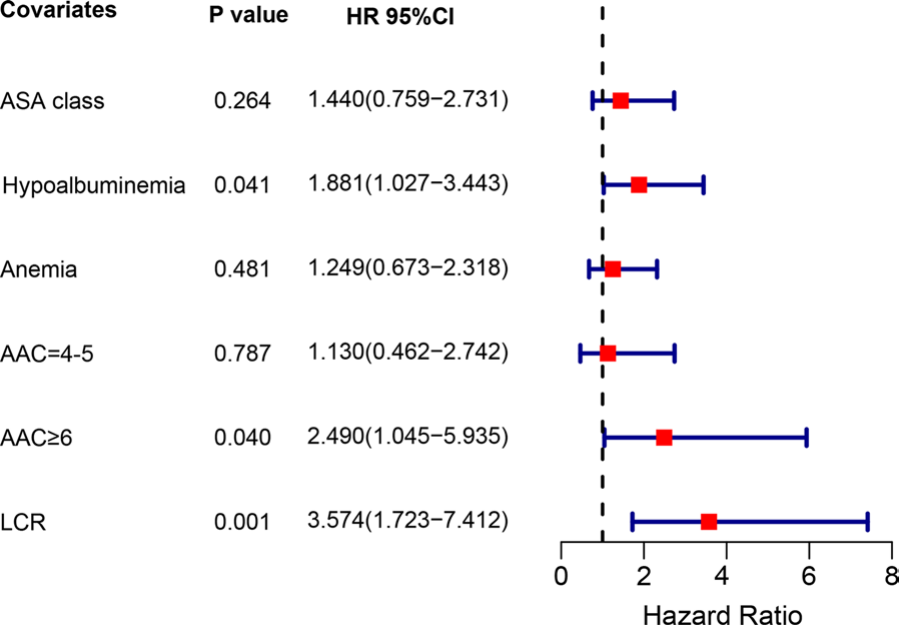
Multivariate Cox regression analysis result of covariates hazard ratio (HR) for 1-year mortality in elderly patients with displaced femoral neck facture undergoing hemiarthroplasty. The red squares represent HRs, and the purple lines represent 95% confidence intervals. *ASA* American Society of Anesthesiologists, *AAC* Age-Adjusted Charlson Comorbidity, *LCR* lymphocyte-to-C-reactive protein ratio

**Fig. 4:**
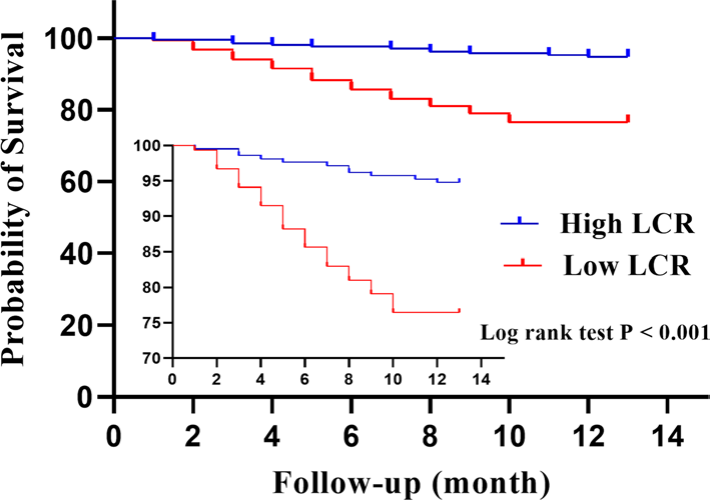
Kaplan–Meier survival curves stratified according to LCR cutoff of 30,560. There was a significant increase in 1-year mortality in patients with preoperative LCR ≤ 30,560 (*P* < 0.001). *LCR* Lymphocyte-to-C-reactive protein ratio

The association between clinical variables and 1-year mortality was shown in Tables 3 and 4. There was no statistical significance found between the survival group and death group regarding postoperative pain improvement and activity of patients, indicating that the death was not related to the effect of replacement surgery. Univariate Cox analysis revealed that greater ASA classification (*P* = 0.045), preoperative anemia (*P* = 0.042), hypoalbuminemia (*P* = 0.001), higher AAC score (*P* < 0.001), and low LCR level (*P* < 0.001) were significantly associated with 1-year mortality. Further multivariate Cox analysis demonstrated that hypoalbuminemia, higher AAC score, and low LCR level were independent predictors for mortality (Fig. 3). Consistent with this result, Kaplan–Meier survival curves stratified by preoperative LCR showed that low LCR was associated with worse 1-year mortality (log-rank test, *P* < 0.001) (Fig. 4).

**Table 3:** Pain levels and preoperative and postoperative mobility according to survival

Variable	Survival group (*n* = 317)	Death group (*n* = 47)	*P* value
Prior walking ability, *n* (%)			0.105
No walking aids	271 (85.5)	35 (74.5)	
1–2 canes/crutches	32 (10.1)	7 (14.9)	
Wheelchair transfer only	14 (4.4)	5 (10.6)	
Pain level			
Opioid medication discontinued, mean (min, max)	2.80 (1–19)	3.28 (1–15)	0.225
Analgesics at discharge, *n* (%)			0.474
Class I analgesics	281 (88.6)	40 (85.1)	
Class II analgesics	19 (6.0)	5 (10.6)	
Class III analgesics	17 (5.4)	2 (4.3)	
Mobility with any walking device, *n* (%)			0.228
Achieved	307 (96.8)	44 (93.6)	
Not achieved	10 (3.2)	3 (6.4)	
Mobility at discharge, *n* (%)			0.158
Independent on crutches	68 (21.5)	8 (17.0)	
No walking aids	120 (37.9)	12 (25.5)	
Walking frame	119 (37.5)	24 (51.1)	
Wheel chair transfer only	10 (3.2)	3 (6.4)

**Table 4:** Univariate analysis of elderly patients with displaced femoral neck facture for 1-year mortality

Variable	HR	95% CI		*P* value
		Lower limit	Upper limit	
Age (≤ 80 vs. > 80)	1.241	0.701	2.199	0.459
Gender (male vs. female)	0.783	0.432	1.420	0.421
Affected side (left vs. right)	1.096	0.619	1.942	0.753
Tobacco consumption (no vs. yes)	2.277	0.817	6.344	0.116
Alcohol consumption (no vs. yes)	1.993	0.483	8.217	0.340
Time to surgery (≤ 48 vs. > 48)	1.144	0.613	2.139	0.672
Prosthesis type (cement vs. cementless)	1.174	0.652	2.114	0.593
Type of anesthesia (general vs. spinal/epidural)	0.782	0.434	1.408	0.412
ASA class (I–II vs. III–IV)	1.825	1.014	3.287	0.045
Hypoalbuminemia (no vs. yes)	2.615	1.467	4.663	0.001
Anemia (no vs. yes)	1.818	1.023	3.231	0.042
AAC				< 0.001
AAC = 1–3	Reference			
AAC = 4–5	1.991	0.903	4.386	0.088
AAC ≥ 6	5.178	2.437	11.002	< 0.001
LCR level (high level vs. low level)	4.98	2.534	9.786	< 0.001

*HR* Hazard ratio, *CI* confidence interval, *ASA* American Society of Anesthesiologists, *AAC* Age-Adjusted Charlson Comorbidity, *LCR* lymphocyte-to-C-reactive protein ratio

### Postoperative complications

In our cohort, 24 (11.4%) patients in the high-LCR group and 30 (19.6%) in the low-LCR group experienced at least one complication after hemiarthroplasty, and the difference was statistically significant (*P* = 0.029) (Table 5). Further analysis of various complications demonstrated that the incidence of various complications was higher in the low-LCR group than in the high-LCR group, although there was no statistically significant difference. In addition, the low-LCR group had higher rate of red blood cell transfusion (*P* = 0.027) and longer hospital stays (*P* = 0.031) compared with high-LCR group.

**Table 5:** Postoperative complications after hemiarthroplasty

Variable	Total (*n* = 364)	Low LCR (*n* = 153)	High LCR (*n* = 211)	*P* value
Total complications (%)	54 (14.8)	30 (19.6)	24 (11.4)	0.029
Urinary tract infection (%)	14 (3.8)	8 (5.2)	6 (2.8)	0.243
Wound infection (%)	7 (1.9)	5 (3.3)	2 (0.9)	0.112
Pneumonia (%)	15 (4.1)	9 (5.9)	6 (2.8)	0.15
Stroke (%)	4 (1.1)	2 (1.3)	2 (0.9)	0.745
Congestive heart failure (%)	4 (1.1)	3 (2.0)	1 (0.5)	0.179
Deep vein thrombosis (%)	10 (2.7)	6 (3.9)	4 (1.9)	0.243
Transfusion (%)	115 (31.6)	58 (37.9)	57 (27.0)	0.027
Length of stay (days)	7.53 ± 2.36	7.84 ± 2.40	7.30 ± 2.32	0.031

*LCR* Lymphocyte-to-C-reactive protein ratio

## Discussion

To the best of our knowledge, this study is the first study to investigate the use of LCR for predicting mortality of elderly patients undergoing hemiarthroplasty for displaced FNF. The most important finding of our study was that low preoperative LCR was an independent predictor for mortality within 1-year after surgery. The ideal cutoff value for preoperative LCR was 30,560 in our cohort. Evaluating preoperative LCR could be helpful to identify elderly FNF patients at high risk for mortality and facilitate individualized perioperative therapeutic strategies. Patients whose preoperative LCR was less than the cutoff value could receive early interventions to improve the survival rate.

LCR has been investigated in many diseases recently, including malignancy, inflammation, and even the coronavirus disease 2019 [14, 21, 22]. However, there was no study focusing on the relationship between LCR and mortality in elderly patients undergoing hemiarthroplasty for displaced FNF. Since both lymphocyte and CRP are influenced by numerous conditions, potential bias may be reduced by using of LCR. In our study, the ROC curve analysis demonstrated that the AUC for LCR (0.686) was higher than lymphocyte count (0.610) and CRP (0.660), which indicated that the prognostic value of LCR is better than either lymphocyte or CRP alone in predicting 1-year mortality in elderly patients with FNF undergoing hemiarthroplasty.

Previous studies have identified that increased CRP had a significant association with trauma, inflammation, and infection [13]. Kim et al. [23] reported elevated CRP on admission was a predictor of 1-year mortality in patients with surgically treated hip fracture. On the other hand, peripheral blood lymphocytes could be used to assess immune–nutrition status of patients. Wilson [24] reported 62.6% of patients with hip fracture were diagnosed as malnutrition which was defined by TLC < 1500 /mm^3^. In addition, the depletion of peripheral blood lymphocytes could also reflect the intensity and strength of stressful events in trauma [25]. In a recent meta-analysis and systematic review, Li [26] showed lower lymphocyte counts were significantly associated with poor survival of elderly patients with surgically treated hip fracture. When combining lymphocytes and CRP, we found that LCR was more prognostic than either CRP or lymphocytes in isolation for elderly patients with surgical treated FNF. We considered that decreased LCR level by lowering lymphocyte counts and/or raising CRP may contribute to the worse mortality. In the current study, patients with low LCR level (≤ 30,560) had a mortality rate of more than 3 times compared to the patients with high LCR level. Therefore, more attention should be paid to the patients with low level of preoperative LCR.

There are several advantages in the clinical utility of LCR. Because of easily calculated from blood test at admission, the LCR is readily available, simplified, and inexpensive. Moreover, it was an objective method to inform clinical decision-making and make a good distinction of patients with different risks. Our study successfully validated the clinical feasibility of LCR with constant cutoff value for predicting mortality in elderly patients undergoing hemiarthroplasty, which will be conducive to the clinical practice. First, accurate prognostic prediction would be helpful to stratify patients and prioritize high-risk patients for orthogeriatric care, which has been proven to be beneficial in reducing mortality in elderly patients with hip fracture [27–29]. Second, unlike other prognostic predictors such as advanced age or AAC that based on unmodifiable patient status, LCR can be changed in the process of treatment and be affected by therapeutic interventions [14]. Therefore, enhancing LCR, such as nutritional support, may be a potentially effective strategy to reduce mortality in elderly patients with surgical treated FNF. However, it needs to be validated in future work.

In our study, we found the patients with low level of preoperative LCR had higher risk of developing postoperative complications, red blood cell transfusion, and longer hospital stay. Among of them, the higher rate of transfusion deserves special mention. Previous studies have reported that allogeneic transfusion was a predictor for 1-year mortality in elderly patients with surgically treated FNF [30]. In addition, hypoalbuminemia and AAC ≥ 6 were also identified as independent risk factors (*P* = 0.041, *P* = 0.040, respectively) for 1-year mortality in multivariate analysis, which was consistent with previous studies [8, 13]. Both of them are known to be associated with vulnerable physiologic state [31]. In general, such patients always fail to follow clinician’ instructions for rehabilitation after surgery and therefore have an increased risk of postoperative complications and mortality. Accordingly, we suggest relevant protocols of prevention should be provide to this population as early as possible to lower the mortality rate and improve quality of life after surgery.

Although low level of preoperative LCR has prognostic value for increased mortality in our cohort, the exact mechanisms are still not clear. We supposed it could be explained by following factors. First, systemic inflammation has been proved to be associated with prognosis in patients with hip fracture [32], and LCR can well reflect the status of systemic inflammation [33]. Second, frailty has been identified as predictor for mortality following femoral neck fracture [34], while low LCR was closely correlated with physiologically vulnerable state of elderly patients [35]. Third, in our study, low LCR was correlated with adverse events after surgery, including pneumonia, deep vein thrombosis, and cardiovascular and cerebrovascular complications. These factors are significantly associated with worse prognosis in hip fracture patients [36, 37]. Taken together, LCR was correlated with systemic inflammation, vulnerability of patients, and postoperative complications, likely influencing the mortality of elderly patients with FNF undergoing hemiarthroplasty.

This study has several limitations. First, inherent shortcomings of the retrospective study design and limited sample size may cause data bias to a certain extent. Second, other factors that may have influenced postoperative survival in elderly FNF patients were not investigated, with possibility of residual confounding. Third, our study only included Chinese patients, while the relationship between racial differences and inflammatory responses has been reported in previous literature [38]. Thus, the generalizability of our results to other races requires to be further validate. Finally, some inflammatory markers with clearly association with prognosis, such as interleukin (IL)-6 and IL-10 [39], were not examined in our study. Therefore, further high-quality researches with prospective design and multicenter participation are needed to verify our findings. Nevertheless, as far as we know, the current study is the first study to report that preoperative LCR is significantly associated with 1-year mortality and complications after surgery in elderly patients undergoing hemiarthroplasty for displaced FNF.

## Conclusions

In conclusion, the level of preoperative LCR can effectively predict 1-year mortality and postoperative complications in elderly patients with displaced FNF undergoing hemiarthroplasty and had better predictive value than lymphocyte count and CRP. Patients with low level of LCR could benefit from a more intensified care and rigorous follow-up strategies. Future work is warranted to determine whether enhancing LCR will decrease mortality for these patients.

## Data Availability

The data contributing to this article may be made available upon request by sending an e-mail to the first author.
